# Automatic detection of fish and tracking of movement for ecology

**DOI:** 10.1002/ece3.7656

**Published:** 2021-05-18

**Authors:** Sebastian Lopez‐Marcano, Eric L. Jinks, Christina A. Buelow, Christopher J. Brown, Dadong Wang, Branislav Kusy, Ellen M. Ditria, Rod M. Connolly

**Affiliations:** ^1^ Coastal and Marine Research Centre Australian Rivers Institute School of Environment and Science Griffith University Gold Coast QLD Australia; ^2^ Quantitative Imaging Research Team CSIRO Marsfield NSW Australia; ^3^ Data61 CSIRO Pullenvale QLD Australia

**Keywords:** computer vision, connectivity, deep learning, dispersal, machine learning, object tracking, underwater video

## Abstract

1. Animal movement studies are conducted to monitor ecosystem health, understand ecological dynamics, and address management and conservation questions. In marine environments, traditional sampling and monitoring methods to measure animal movement are invasive, labor intensive, costly, and limited in the number of individuals that can be feasibly tracked. Automated detection and tracking of small‐scale movements of many animals through cameras are possible but are largely untested in field conditions, hampering applications to ecological questions.
2. Here, we aimed to test the ability of an automated object detection and object tracking pipeline to track small‐scale movement of many individuals in videos. We applied the pipeline to track fish movement in the field and characterize movement behavior. We automated the detection of a common fisheries species (yellowfin bream, *Acanthopagrus australis)* along a known movement passageway from underwater videos. We then tracked fish movement with three types of tracking algorithms (MOSSE, Seq‐NMS, and SiamMask) and evaluated their accuracy at characterizing movement.
3. We successfully detected yellowfin bream in a multispecies assemblage (F1 score =91%). At least 120 of the 169 individual bream present in videos were correctly identified and tracked. The accuracies among the three tracking architectures varied, with MOSSE and SiamMask achieving an accuracy of 78% and Seq‐NMS 84%.
4. By employing this integrated object detection and tracking pipeline, we demonstrated a noninvasive and reliable approach to studying fish behavior by tracking their movement under field conditions. These cost‐effective technologies provide a means for future studies to scale‐up the analysis of movement across many visual monitoring systems.

## Graphical abstract

We applied automatic object detection and object tracking to track fish movement in the field and characterise movement behaviour.

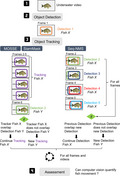

## 1 INTRODUCTION

Computer vision, the research field that explores the use of computer algorithms to automate the interpretation of digital images or videos, is revolutionizing data collection in science (Beyan & Browman, 2020; Waldchen & Mader, 2018). The use of remote camera imagery, such as underwater stations, camera traps, and stereography, has driven the uptake of computer vision because it can process and analyze imagery quickly and accurately (Bicknell et al., 2016; Schneider et al., 2019). In ecological studies, advances in computer vision have led to increased sampling accuracy and repeatability (Waldchen & Mader, 2018). For example, drones are being used to track grassland animals (van Gemert et al., 2015) and estimate tree defoliation (Kälin et al., 2019), underwater observatories with computer vision are monitoring deep‐sea ecosystems (Aguzzi et al., 2019), and computer vision‐capable dive scooters are being used to monitor coral reefs at large spatial and temporal scales (González‐Rivero et al., 2020; Kennedy et al., 2020).

In the past few years, we have seen an increase in the uptake of computer vision to study and monitor marine ecosystems. These applications are related to the two main computer vision tasks: object detection and object tracking. Object detection and object tracking automate data collection, including gathering information about the type, location, and movement of objects of interest. Object detection algorithms can count and identify species of interest in underwater video footage (Christin et al., 2019) and have been applied to detect seals (Salberg, 2015), identify whale hotspots (Guirado et al., 2019), monitor fish populations (Ditria, Lopez‐Marcano, et al., 2020; Jalal et al., 2020; Marini et al., 2018; Salman et al., 2016; Villon et al., 2016, 2018, 2020; Xiu et al., 2015), and quantify floating debris on the ocean surface (Watanabe et al., 2019). On the other hand, object tracking can locate and output the movement direction and speed of objects between video frames. In marine ecosystems, object tracking has been used to track on‐surface objects (see topios.org) and underwater objects such as fish, sea turtles, dolphins, and whales (Arvind et al., 2019; Chuang et al., 2017; Kezebou et al., 2019; Spampinato et al., 2008; Xu & Cheng, 2017).

There is evidence that automated monitoring of fish in underwater ecosystems through the combination of object detection and object tracking is reliable and accurate (Lantsova et al., 2016; Mohamed et al., 2020; Spampinato et al., 2008). However, no studies have jointly applied object detection and object tracking for animal movement studies. Object detection can automatically collect traditional presence/absence data of different species (Marini et al., 2018; Xiu et al., 2015) while object tracking simultaneously tracks individuals to provide fine‐scale data to assess behavioral and animal movement patterns (Francisco et al., 2020). Combining object detection and tracking in a single and noninvasive automated approach enhances the amount of ecologically relevant information extracted from videos. This subsequently improves, for example, our ability to quantify and evaluate environmental drivers of species abundance, diversity, movement, and behavior.

The utility of combining object detection and tracking is particularly useful for studying animal movement, which typically requires large volumes of data for many individuals (Librán‐Embid et al., 2020; Lopez‐Marcano et al., 2020). Knowledge of animal movement is fundamental to many research objectives in marine science, as animal movement shapes predator–prey dynamics, nutrient dynamics, and trophic functions (Olds et al., 2018). For example, the movement of herbivorous fish between seagrass and coral reefs helps maintain resilience by balancing fish abundances with algal growth rates that vary spatio‐temporally (Pagès et al., 2014). Collecting movement data is, however, challenging and requires substantial resources. The development and applications of automated technologies (i.e., object detection and tracking pipelines) can overcome these restrictions and help advance our understanding of animal movement across a broad range of spatio‐temporal scales and ecological hierarchies (e.g., individuals, populations, communities).

In this study, we aimed to test the ability of deep learning algorithms to track small‐scale animal movement of many individuals in underwater videos. We developed a computer vision pipeline consisting of two steps, object detection and object tracking, and we used the pipeline to quantify underwater animal movement across habitats. To demonstrate the benefits of combining object detection and object tracking, we deployed cameras in a known coastal fish estuarine passageway and recorded the movement of a common fisheries species (yellowfin bream, *Acanthopagrus australis)*. Ultimately, we demonstrate that these technologies can complement the collection and analysis of animal movement data and potentially contribute to the data‐driven management of ecosystems.

## 2 METHODS

### 2.1 Object detection

Object detection is a field of computer vision that deals with detecting instances of objects in images and videos (Zhao et al., 2019). Methods for object detection generally include traditional image processing and analysis algorithms and deep learning techniques (Zhao et al., 2019). Deep learning is a subset of machine learning that uses networks capable of learning higher dimensional representations and detect patterns within unstructured data (Lecun et al., 2015; Schmidhuber, 2015). In this paper, we used deep learning, and more specifically Mask Regional Convolutional Neural Network (Mask R‐CNN) for fish detection (Cui et al., 2020; Ditria, Lopez‐Marcano, et al., 2020; Jalal et al., 2020; Villon et al., 2020). Mask R‐CNN is one of the most effective open‐access deep learning models for locating and classifying objects of interest (He et al., 2017).

To develop and train the fish detection model, we collected video footage of bream in the Tweed River estuary, Australia (−28.169438, 153.547594) between May and September 2019. We used six submerged action cameras (1080p Haldex Sports Action Cam HD) deployed for 1 hr in a variety of marine habitats (e.g., rocky reefs and seagrass meadows). We varied the camera angle and placement to capture diverse backgrounds and fish angles (Ditria et al., 2020). We trimmed the original 1‐hr videos into snippets where bream were present using VLC media player 3.0.8. The snippets were then converted into still frames at 5 frames per second. The training videos included 8,700 fish annotated across the video sequences (Supplementary A). We used software developed at Griffith University for data preparation and annotation tasks (FishID—https://globalwetlandsproject.org/tools/fishid/). We trained the model using a ResNet50 architecture with a learning rate of 0.0025 (He et al., 2017). We used a randomly selected 90% sample of the annotated dataset for the training, with the remaining 10% for validation. To minimize overfitting, we used the early‐stopping technique (Prechelt, 2012), where we assessed mAP50 on the validation set at intervals of 2,500 iterations and determined where the performance began to drop. mAP50 is a measurement of the model's capacity to overlap a segmentation mask around 50% of the ground‐truth outline of the fish (Everingham et al., 2010). We used a confidence threshold of 80%, meaning that we selected object detection outputs where the model was 80% or more confident that it was a bream. We developed the models and analyzed the videos using a Microsoft Azure Data Science Virtual Machine powered with either NVIDIA V100 GPUs or Tesla K80 GPUs.

### 2.2 Object tracking

**FIGURE 1:**
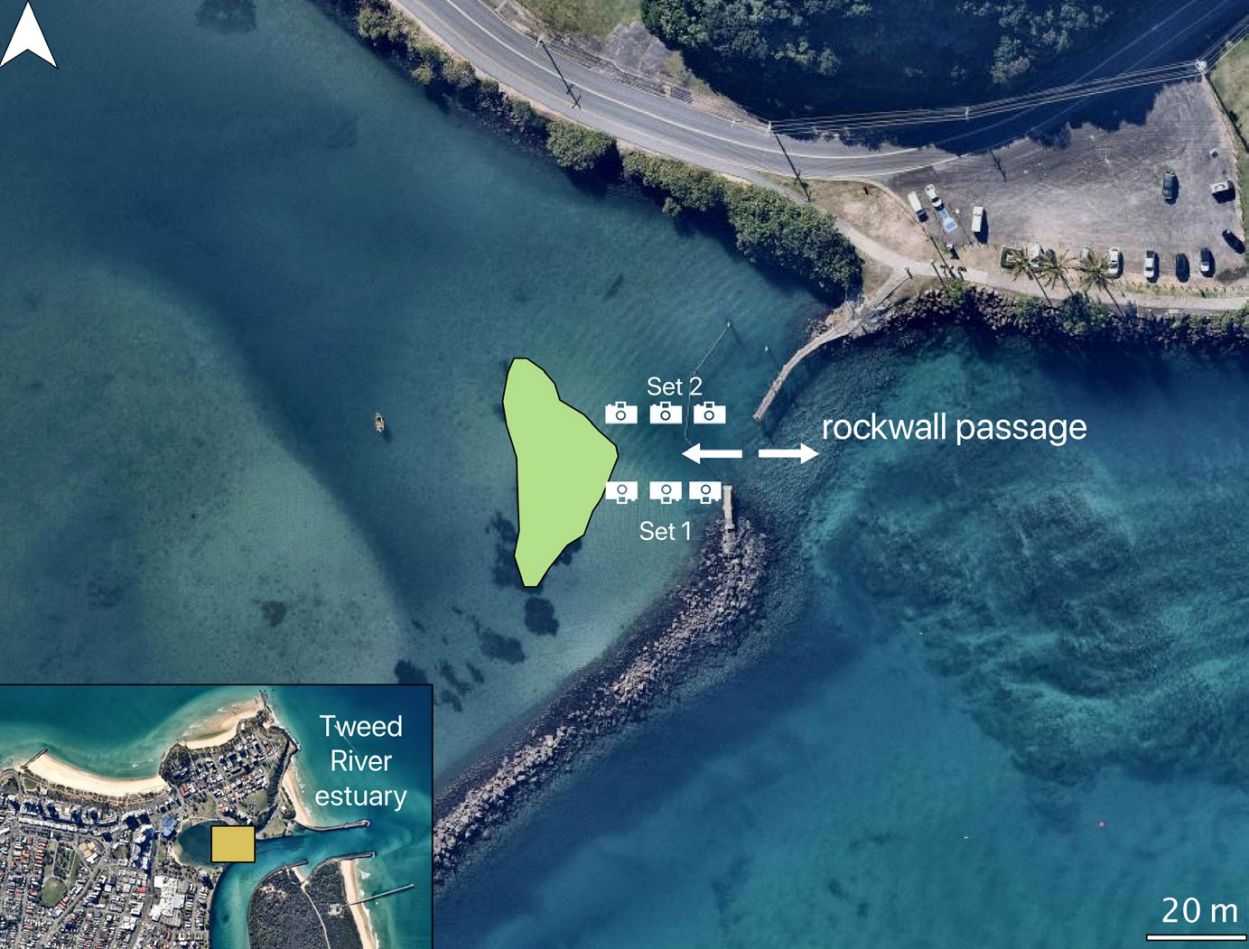
The study location in Tweed River Estuary, Australia, showing the camera array deployed in a fish passageway (two ended white arrow) between the rock wall channel and the seagrass meadow (green polygon). Each set of cameras consisted of three underwater cameras that recorded for 1 hr during a flood tide. Set 1 faced north and set 2 faced south. The distance between cameras (~3 m) and between sets (20 m) ensured nonoverlapping field of views. Map data: NearMap 2020

Tracking objects in underwater videos is challenging due to the 3D medium that aquatic animals move through, which can be obscured by floating objects, and which creates greater variation in the shape and texture of the objects and their surroundings in a video (Sidhu, 2016). Advances in object tracking are addressing these issues, and objects can now be tracked consistently despite natural variations of the object's shape, size, and location (Bolme et al., 2010; Cheng et al., 2018). We developed a pipeline where the object tracking architecture activated once the object detection model detected a bream. This approach resulted in an automated detection and subsequent tracking of fish from the underwater videos. Additionally, we benchmarked the performance of three object tracking architectures: minimum output sum of squared errors (MOSSE) (Bolme et al., 2010), sequential nonmaximum suppression (Seq‐NMS) (Han et al., 2016), and Siamese mask (SiamMask) (Wang et al., 2019) by using movement data gathered one month after the training dataset was collected from a different location in the Tweed River estuary, Australia. In this location, a 150‐m long rocky wall restricts access to a seagrass‐dominated harbor (Figure 1). The placement of the rock wall creates a 20‐m wide passageway that fish use as a movement corridor to access a seagrass meadow. Multiple species of estuarine fish such as sand whiting (*Sillago ciliata),* river garfish (*Hyporhamphus regularis*), luderick (*Girella tricuspidata)*, spotted scat (*Scatophagus argus*), three‐bar porcupinefish (*Dicotylichthys punctulatu*s), and bream, move back and forth with the tides through this passageway. This environment suffers from frequent low visibility and currents that bring floating debris, presenting a relatively challenging scenario in which to showcase the capacity of computer vision to detect the target species in a multispecies assemblage and quantify the direction of movement.

We collected fish movement data by submerging two sets of three action cameras (1080p Haldex Sports Action Cam HD) for 1 hr during a morning flood tide in October 2019. We placed the sets of cameras parallel to each other and separated by 20 m (Figure 1). Within each set, the cameras faced horizontally toward the fish corridor and parallel with the seafloor and were separated by ~3 m. The camera placement allowed us to calculate horizontal movement (left or right) of fish through the corridor. The distance between the cameras and between the sets ensured nonoverlapping field of views. Set 1 cameras faced north and Set 2 faced south (Figure 1). We placed the cameras in a continuous line starting at the harbor entrance and ending at the border of the seagrass meadow, deployed at a depth of 2–3 m. We manually trimmed each video using VLC media player 3.0.8 into video snippets with continuous bream movement, resulting in 76 videos of varying durations (between 3 and 70 s), which we converted into still frames at 25 frames per second. All frames with bream were manually annotated, and these annotations were used as ground‐truth. We used the fish movement dataset to evaluate the object detection model and the object tracking architectures.

#### 2.2.1 Minimum output sum of squared error (MOSSE)

**FIGURE 2:**
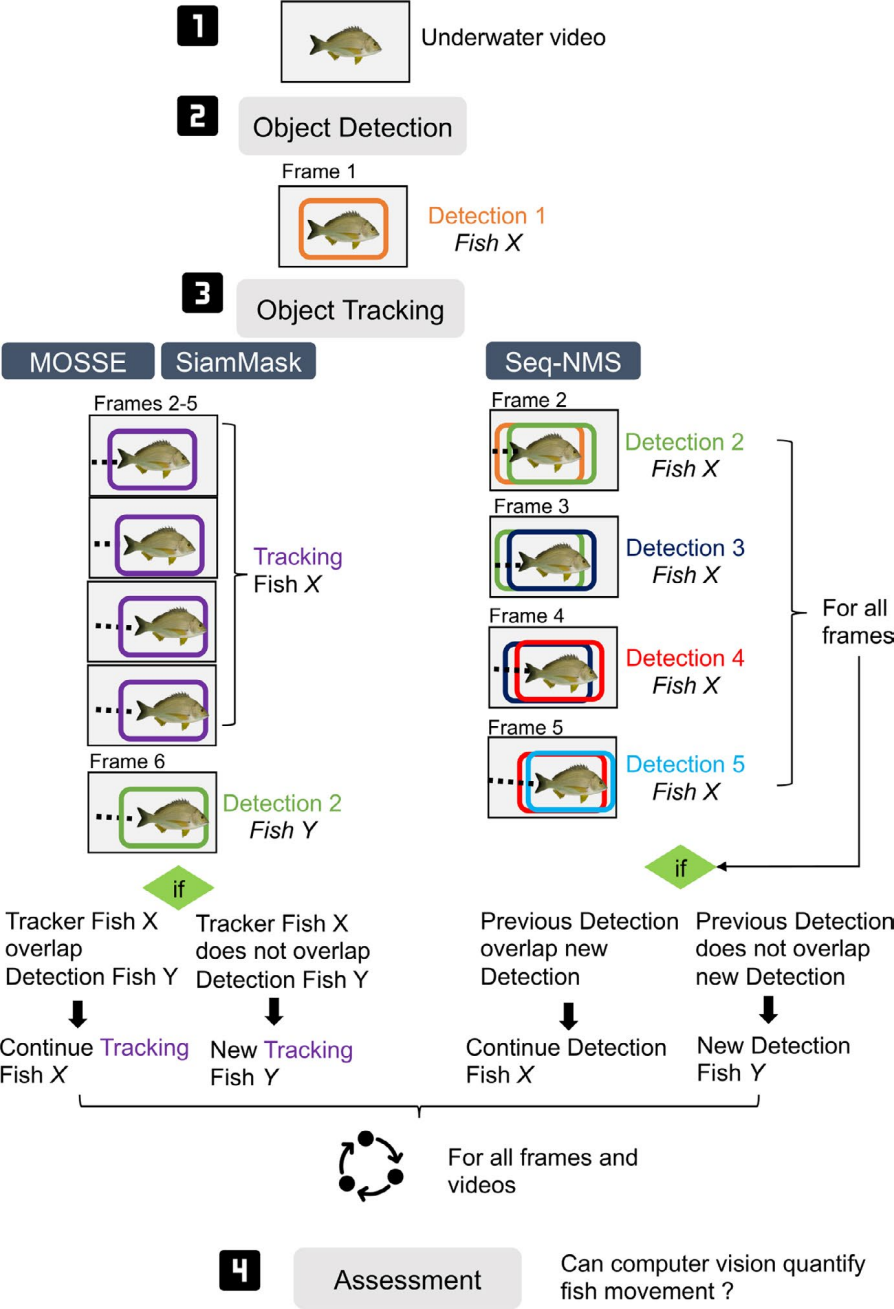
Interaction between the object detection model and tracking architectures. The object detection model activates all three tracking architectures. For MOSSE and SiamMask, the tracker continues for 4 frames after the initial detection. For Seq‐NMS, the movement was determined by calculating the vector direction between two detections. For all architectures, a check was made to determine if the tracker continued, stopped, or a new tracker started. For MOSSE and SiamMask, the check was made after 4 tracking frames from the first detection. For Seq‐NMS, the check was made for all frames after the first detection. The interaction between detections and tracker occurred through the whole length of a video where the object detection model detected a yellowfin bream and was carried for all frames, videos, and cameras. All trackers provided a direction of movement for each frame where the interaction between the detection and tracking occurred successfully

The MOSSE algorithm produces adaptive correlation filters over target objects, and tracking is performed via convolutions (process of combining outputs to form more outputs). MOSSE was developed between 2010 and 2016 and is robust to changes in lighting, scale, pose, and shape of objects (Bolme et al., 2010; Sidhu, 2016). Here, we modified the MOSSE tracking process by activating the tracker with the object detection output (Figure 2). The object detection model and the object tracking architecture interacted to maintain the consistency of the tracker on bream individuals. When a fish was detected, the entry was used to initialize the tracker. MOSSE tracked the fish for four frames, and a check was made on the subsequent frame to verify the accuracy of the tracker. In this check, if the detection bounding box overlapped by ≥30% with the existing tracker bounding box, the tracker continues on the same object. If the detection bounding box does not overlap with the existing tracker bounding box, a new tracker entry starts. This interaction between the detection and tracking occurred for every fish detected in a frame and stopped when no more detections were found.

#### 2.2.2 Sequential nonmaximum suppression (Seq‐NMS)

Sequential nonmaximum suppression (Seq‐NMS) was developed in 2016 traditionally to improve the classification results and consistency of deep learning outputs (Han et al., 2016). Seq‐NMS works differently to the other trackers tested because it requires an object detection output for every frame containing a fish. Seq‐NMS links detections of neighboring frames, which means that a detection in the first frame can be connected with a detection in the second frame if there is an intersection above a defined threshold. In our case, we used the principles of Seq‐NMS to create detection linkages for object tracking of fish when there was an overlap (intersection over union) of bounding boxes in subsequent frames of ≥30% (Figure 2). If this is true, then the chain of detections continues. When the overlap is less than 30%, then a new detection link starts (i.e., the tracker will treat this detection as a new fish).

#### 2.2.3 SiamMask

SiamMask is a tracking algorithm developed in 2019 that uses outputs of deep learning models for estimating the rotation and location of objects (Wang et al., 2019). SiamMask is based on the concepts of Siamese network‐based tracking. Similar to MOSSE, we slightly modified the tracking process by activating the tracker with the deep learning object detection model (Figure 2). The tracking with SiamMask started once a bream was detected (Figure 2).

We have made all object detection annotations, images, trackers, and data wrangling codes, as well as the movement dataset openly available (https://doi.org/10.5281/zenodo.4571760).

### 2.3 Model evaluations and movement assessment

#### 2.3.1 Object detection evaluation

We evaluated the object detection against the movement data (manually annotated and ground‐truthed) described in section 2.2 and calculated precision, recall, and F1. The precision is the rate of true positives relative to total detections, and the recall is the rate of detection of true positives. We used the F1 score (the harmonic mean of the precision and recall) to assess the performance of our object detection model in answering ecological questions on abundance.

(1) $$\text{Precision}=\frac{\text{True Positives}}{\text{True Positives}+\text{False Positives}}$$

(2) $$\text{Recall}=\frac{\text{True Positives}}{\text{True Positives}+\text{False Negatives}}$$

(3) $$F1=2*\frac{\text{precision}*\text{recall}}{\text{precision}+\text{recall}}$$

Additionally, we determined the model's ability to fit a segmentation mask around the fish through the mean average precision value (mAP) (Everingham et al., 2010). We used the mAP50 value, which is the model's capacity to overlap a segmentation mask around 50% of the ground‐truth outline of the fish. A high mAP50 value means that the model has high accuracy when overlapping a mask around the fish. We used the COCO evaluation python script to calculate mAP50 (Massa & Girshick, 2018).

#### 2.3.2 Object tracking evaluation

We evaluated the tracking architectures against the movement dataset by calculating precision, recall, and an F1 score and by assessing the movement data. To calculate precision recall and an F1 score, we manually observed every second of video and determined if the object tracking architecture was correctly tracking the bream individual (Supplementary B). We defined a true positive as a correct detection and accurate tracking of the individual for ≥50% of the time where bream appeared on frame (Supplementary B). A false negative occurred when a bream was not detected and tracked or if it was tracked <50% of the time when the fish appeared on frame. Additionally, we classified a false positive when a nonbream object was detected and tracked, or when a bream was detected but the tracking architecture tracked a nonbream object.

#### 2.3.3 Movement assessment

We conducted a movement assessment to evaluate the accuracy of the directions provided by the tracker. From all trackers, we obtained the bounding boxes and centroids of the boxes for fish that were detected and subsequently tracked. For each tracking output, the object tracking architecture provided a tracking angle of movement in 2 dimensions relative to the camera frame. Depending on the camera set (Figure 1), we summarized angles for north‐facing cameras (Set 1) and south‐facing cameras (Set 2). We grouped tracking angles using reference angles into four directions: up, down, left, and right (Supplementary B). Because the cameras were facing horizontally toward the fish passageway parallel with the seafloor, we calculated horizontal movement of fish. Fish moving up meant that the fish movement had tracking angles between 0°–44° and 315°–360°. Fish moving right had angles between 45° and 135°, whereas fish moving left between 225° and 315°. Finally, fish moving down had tracking angles between 135° and 225°. The tracking angle for all object tracking architectures was obtained from the tracker vector generated within each tracker's bounding box (Supplementary B). By grouping the directions, we can count and group the number of movement angles per camera and per set. For each camera set, we then calculated the proportion of each tracking direction and determined net movement. We defined net movement as the movement angle with the highest proportion for a video. The data summary was generated in R with the packages ggplot and sqldf (Grothendieck, 2017; Wickham, 2009).

To ground‐truth the tracking data, we manually observed all the videos and determined the direction of movement for each fish (fish moving mainly right or left). We determined the net movement of each video (direction with the highest proportion for the video) and compared the ground‐truth output to the net movement direction from the three object tracking architectures (Supplementary B).

## 3 RESULTS

### 3.1 Object detection

Using the Mask R‐CNN framework for detecting bream, we obtained an 81% mAP50 value and an F1 score of 91% (Table 1). The object detection model missed 21 bream (false negatives) and misidentified 8 objects (e.g., algae or other fish) as bream (false positives) out of the 169 fish (ground‐truth) that were observed.

**TABLE 1:** Object detection mAP50 and the evaluation results of the Mask R‐CNN yellowfin bream model. The confusion matrix is shown as counts of individual fish, where the true positives were the correct detection of yellowfin bream. Yellowfin bream not detected were false negatives and misidentified objects were false positives

Task	mAP50	Confusion matrix				Average precision	Average recall	F1
		Ground‐truth	True positives	False positives	False negatives			
Object detection	81%	169	148	8	21	95%	88%	91%

### 3.2 Object tracking

We simultaneously detected and tracked 1 to 30 individual bream per video. All three architectures detected and subsequently tracked more than 120 of the 169 individual fish that swam through the passageway (Table 2). Average precision values for all architectures were above 80%, with Seq‐NMS the most precise at detecting and tracking the bream (93%). Recall among architectures was very similar at around 73%. The architecture with the highest overall success at detecting and tracking bream was Seq‐NMS (F1 = 84%) (Table 2).

**TABLE 2:** Confusion matrix for the three object tracking architectures (MOSSE, Seq‐NMS, and SiamMask) are shown as counts of individual fish, where the true positive means a bream was detected and tracked correctly for ≥50% of the time when it appeared on a video frame, otherwise, it was false negative. False positives were misidentified objects (e.g., algae or other fish) that were detected and tracked

Architecture	Confusion matrix			Average precision	Average recall	F1
	True positives	False positives	False negatives			
MOSSE	123	23	46	84%	73%	78%
Seq‐NMS	129	9	40	93%	76%	84%
SiamMask	121	19	48	86%	72%	78%

### 3.3 Movement assessment

**FIGURE 3:**
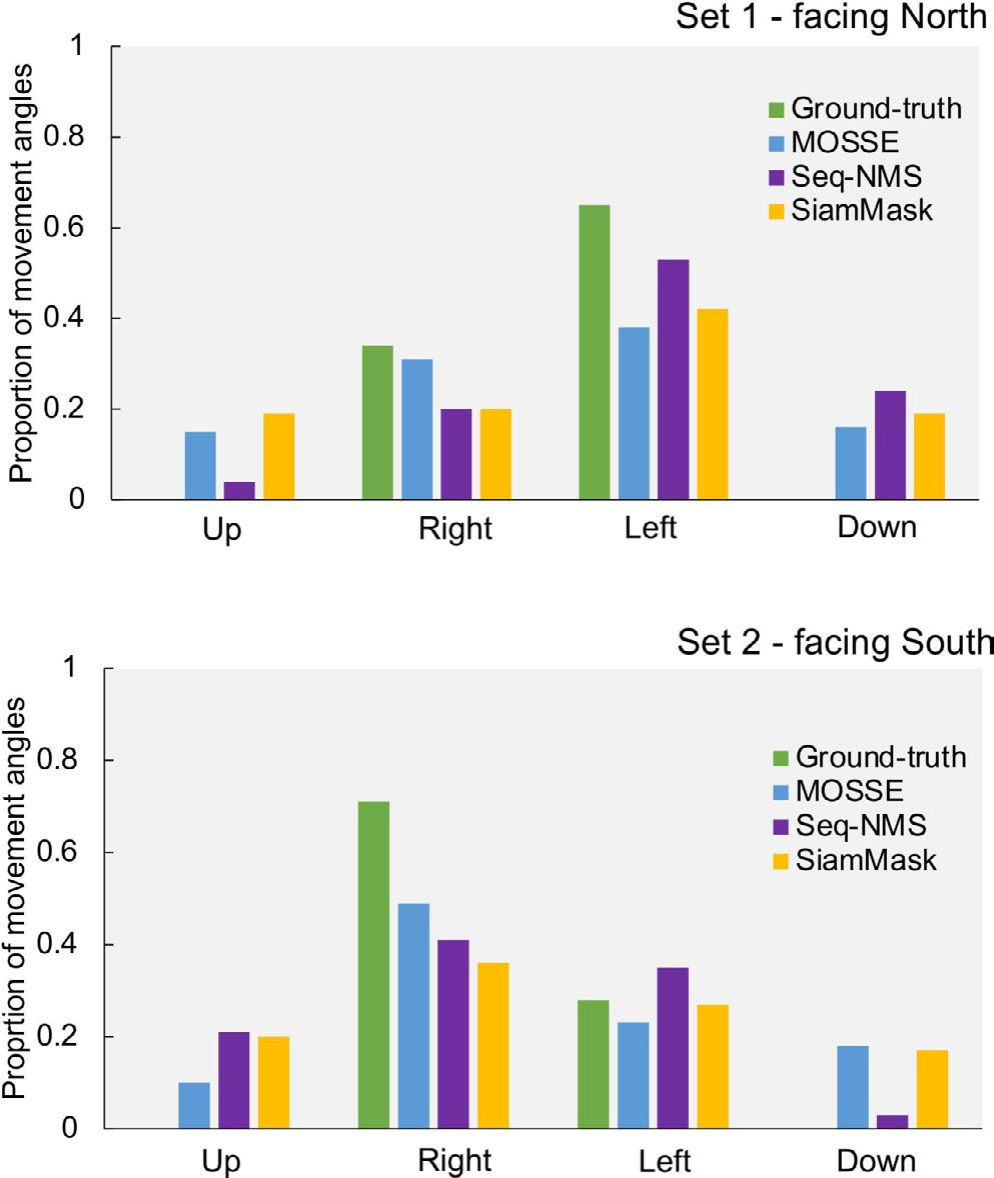
Proportion of the movement angles (up, down, right, left) for the ground‐truth and the three tracking architectures and for the two camera sets (Set 1: facing north and Set 2: facing south). The movement angles are spatial angles of horizontal yellowfin bream movement in two dimensions

We expected the cameras to detect and track fish moving in the passageway consistent with the direction of the tidal flow (i.e., bream moving to seagrass). The expected results were that bream would mostly move to the left (Set 1) and to the right (Set 2), and these patterns were observed when manually analyzing the videos (ground‐truth). The movement direction with the highest proportion for all tracking architectures was left (Set 1) and right (Set 2) (Figure 3). For Set 1, Seq‐NMS (0.53) was the closest to the ground‐truth (0.65), and for Set 2, MOSSE (0.49) and Seq‐NMS (0.41) were the closest to the ground‐truth (0.71) (Figure 3).

## 4 DISCUSSION

We demonstrate a computer vision‐based method for detecting and tracking individual fish in underwater footage. Our study incorporates open‐source computer vision methods into a pipeline that allows scientists to assess animal movement in marine ecosystems. This method quantified animal behavior and detected the expected tidal movement in our case study. The experimental results show that the proposed method is an effective and noninvasive way to detect and track small‐scale movement of many fish in aquatic environments.

Previous ecological work has tracked fish in controlled environments (Bingshan et al., 2018; Papadakis et al., 2014; Qian et al., 2016; Sridhar et al., 2019), used automated detections and counts as proxies for movement (Marini et al., 2018), and, most recently, used automated movement tracking algorithms to quantify movement (Francisco et al., 2020). Automated approaches tested in “real‐world” scenarios provide the best indication and evidence that computer vision is a robust technique for fish monitoring in aquatic ecosystems. When evaluating the object tracking architectures, Seq‐NMS had the best performance and was able to quantify the net movement of multiple individuals. The number of fish that were simultaneously detected and tracked by our framework ranged from 1 to 30 individuals per video. While the movement dataset did not contain videos with a very large number of individuals (e.g., >50 fish in a single frame), previous research has shown that occlusion can influence the accuracy of both the object detection algorithms and Seq‐NMS (Connolly et al., 2021). Moreover, Seq‐NMS is not an object tracking algorithm and it requires a high‐performing object detection model because it uses the object detection outputs of every frame to create the detection links and track the movement direction.

A key benefit of camera‐assisted applications and computer vision analysis to animal movement research, and science more broadly, is that these approaches can complement traditional data collection techniques (Lopez‐Marcano et al., 2020). Cameras and computer vision can be deployed at many sites and cover large spatial extents, but are limited by environmental factors and are incapable of detecting and classifying complex ecological parameters such as predatory interactions or the identification of morphologically similar, but taxonomically different, species (Christin et al., 2019). Traditional approaches (e.g., netting or in‐water diver assessments) are superior at collecting the highest variety and complexity of ecological variables and parameters, but by combining cameras, automation, and traditional approaches, the spatial and temporal scope of monitoring can be increased. Moreover, computer vision approaches do not require specialized equipment to study animal movement and the rapid analysis of imagery can provide movement data that is accurate, valid, and consistent (Francisco et al., 2020; Weinstein, 2018).

The combination of object detection and object tracking can enhance animal movement ecology through the streamlined collection of several sets of ecological information (Botella et al., 2018; Christin et al., 2019), and this new data may revolutionize ecological studies. Traditional presence/absence data can be used, for example, to understand the environmental drivers of a species’ geographic distribution, and the collection of presence/absence data from videos can easily be automated (González‐Rivero et al., 2020; Kennedy et al., 2020; Schneider et al., 2018, 2019). However, presence/absence data alone cannot inform about how multiple ecological processes interact, and presence/absence data conflate movement of individuals with mortality (Zurell et al., 2018). Future studies could use our combined object detection and object tracking approach to simultaneously quantify species distributions and movement. The integration of movement data into species distribution models means that the models could accurately predict how the ranges of mobile species respond dynamically to environmental change through individual movement decisions and population‐level parameters like mortality (Bruneel et al., 2018).

The capacity of our computer vision approach for monitoring fish populations is dependent on the underwater camera setup within the desired seascape. We deployed an array of cameras in a fish passageway to maximize the collection of movement data. However, each set and camera obtained unequal amounts of data and the array also resulted in repeated tracking of fish. Therefore, an important consideration when using camera‐based technologies is to design and deploy an appropriate camera system to monitor animal interactions (Glover‐Kapfer et al., 2019; Wearn & Glover‐Kapfer, 2019). While we demonstrate that the detection and tracking of fish can be automated in aquatic ecosystems, further research into methodological designs (e.g., the optimal number of cameras needed to detect movement) is still required. The development of standardized camera‐based methodologies, such as methodological guides for baited remote underwater surveys (Langlois et al., 2020) or for camera traps (Rovero et al., 2013), but specific to computer vision‐ecology applications will help advance the applications of computer vision into movement ecology.

By utilizing a combination of computer vision frameworks, we demonstrated that automated tracking of fish movement between distinct seascapes (i.e., artificial and natural) is possible. We suggest that these methods are transferable to other types of fish passageways and other habitats, such as the mangrove, seagrass, and coral reef continuum (Francisco et al., 2020; Olds et al., 2018; Spampinato et al., 2008). Further development of these models and architectures, for example, integrated object detection and object tracking with stereo video (Huo et al., 2018) and pairwise comparisons of detections (Guo et al., 2020), will likely lead to improvements in accuracy and for 3D triangulation of detections. Continual improvements in accuracy will provide a rigorous framework to study and quantify fish connectivity in the wild.

## 5 CONCLUSION

Computer vision and automated techniques offer a new generation of methods for collecting and analyzing movement data. Our combined object detection and object tracking approach complements, rather than replaces, traditional techniques. Although current computer vision techniques have limitations, we demonstrated that object detection and object tracking can monitor small‐scale movement of many individuals from underwater footage. The combination of object detection and object tracking has the capacity to provide several streams of ecological information that can inform data‐driven decision that directly influence the health and productivity of marine ecosystems.

## CONFLICTS OF INTEREST

The authors declare that there is no conflict of interest.

## AUTHOR CONTRIBUTIONS

**Sebastian Lopez‐Marcano:** Conceptualization (lead); Data curation (lead); Formal analysis (equal); Funding acquisition (lead); Investigation (lead); Methodology (equal); Project administration (lead); Resources (equal); Software (supporting); Supervision (equal); Validation (lead); Visualization (equal); Writing‐original draft (lead); Writing‐review & editing (lead). **Eric Jinks:** Conceptualization (supporting); Data curation (supporting); Formal analysis (equal); Funding acquisition (supporting); Investigation (supporting); Methodology (equal); Project administration (supporting); Resources (supporting); Software (lead); Supervision (supporting); Validation (supporting); Visualization (supporting); Writing‐original draft (equal); Writing‐review & editing (equal). **Christina A Buelow:** Conceptualization (supporting); Data curation (equal); Formal analysis (equal); Funding acquisition (supporting); Investigation (supporting); Methodology (supporting); Software (supporting); Supervision (supporting); Visualization (equal); Writing‐original draft (equal); Writing‐review & editing (equal). **Christopher J Brown:** Conceptualization (supporting); Data curation (supporting); Formal analysis (supporting); Methodology (supporting); Supervision (lead); Validation (supporting); Visualization (equal); Writing‐original draft (equal); Writing‐review & editing (equal). **Dadong Wang:** Conceptualization (supporting); Methodology (equal); Resources (equal); Software (equal); Supervision (lead); Writing‐original draft (equal); Writing‐review & editing (equal). **Branislav Kusy:** Conceptualization (supporting); Investigation (supporting); Methodology (supporting); Software (supporting); Supervision (lead); Writing‐original draft (equal); Writing‐review & editing (equal). **Ellen Ditria:** Investigation (supporting); Methodology (supporting); Resources (supporting); Software (supporting); Writing‐original draft (supporting); Writing‐review & editing (equal). **Rod Connolly:** Conceptualization (equal); Data curation (supporting); Formal analysis (supporting); Funding acquisition (supporting); Investigation (supporting); Methodology (supporting); Project administration (supporting); Resources (supporting); Software (supporting); Supervision (lead); Writing‐original draft (equal); Writing‐review & editing (equal).

### OPEN RESEARCH BADGES

This article has earned an Open Data and Open Materials Badge for making publicly available the digitally‐shareable data necessary to reproduce the reported results. The data is available at 10.5281/zenodo.4571757 and https://github.com/slopezmarcano/automated‐fishtracking/tree/fish‐track2.

## Supporting information

Supplementary Material

Supplementary Material

## Data Availability

The training images and annotations, movement dataset annotations, images and videos, and the tracking and data wrangling scripts have been made available at (https://doi.org/10.5281/zenodo.4571760).
