# Supplementary material for: Automatic detection of fish and tracking of movement for ecology
Source: Ecol Evol. 2021 May 18;11(12):8254–63. doi: 10.1002/ece3.7656 (PMC8216886; doi:10.1002/ece3.7656)
Supplement: Supplementary file 1 — Supplementary Material [file ECE3-11-8254-s002.docx]

**Appendix S1 Training for object detection model**

To develop the OD model, we collected video footage of the target species, yellowfin bream in the Tweed River estuary, Australia (-28.169438, 153.547594) between May and September 2019. Through each video, we manually annotated 8,700 fish to train the OD model. We annotated bream at different angles, backgrounds and water turbidity (Fig. 1). We used software developed at Griffith University for data preparation and annotation (FishID - https://globalwetlandsproject.org/tools/fishid/).

| 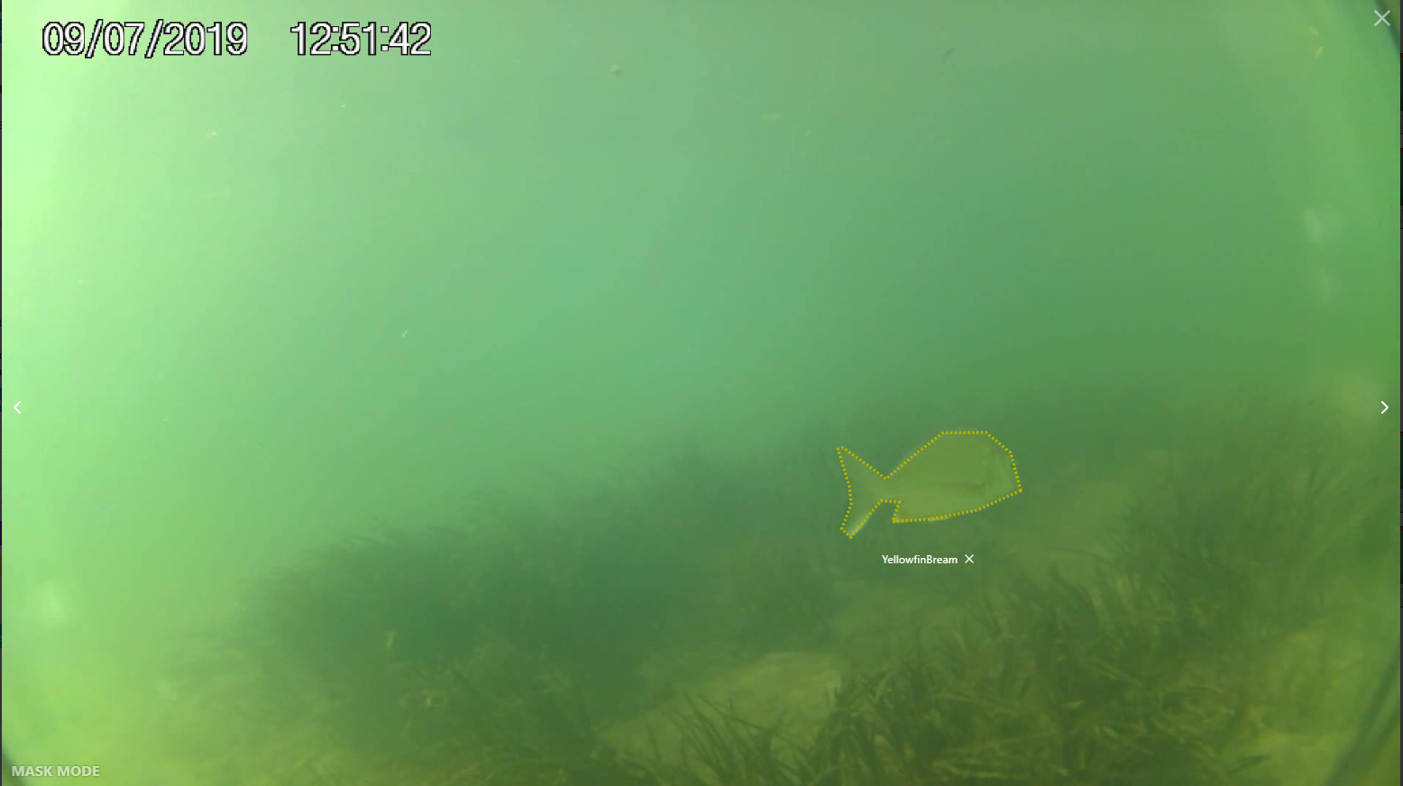 | *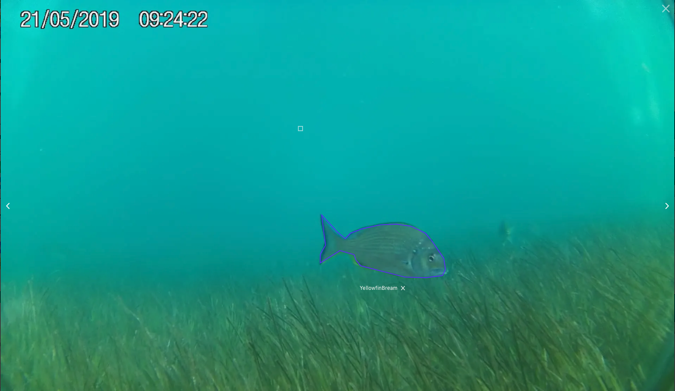* |
| --- | --- |
| *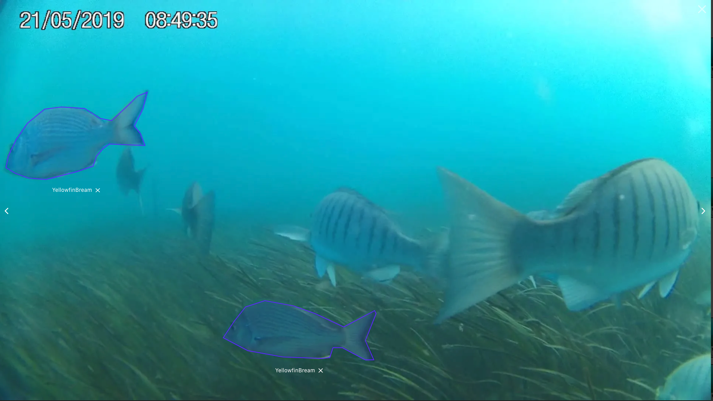* | *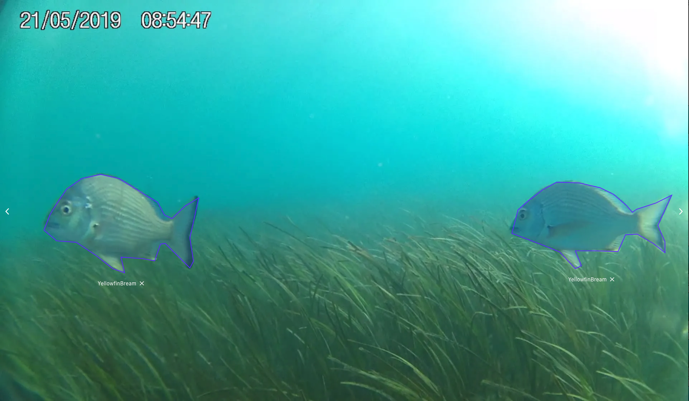* |

**FIGURE 1** Video frame samples used for the training of the Mask R-CNN model, including manually drawn segmentation masks (yellow or blue dotted line) around individual yellowfin bream across different environmental conditions.
