# Supplementary material for: Automatic detection of fish and tracking of movement for ecology
Source: Ecol Evol. 2021 May 18;11(12):8254–63. doi: 10.1002/ece3.7656 (PMC8216886; doi:10.1002/ece3.7656)
Supplement: Supplementary file 2 — Supplementary Material [file ECE3-11-8254-s001.docx]

**Appendix S2 Fish tracking visualisations**

We developed a computer vision pipeline of two tasks, object detection (OD) and object tracking (OT) to assess movement of a fisheries species in a known coastal fish corridor. The OT architecture activated once the OD model detected a fish of the target species (yellowfin bream). This approach resulted in an automated detection and subsequent tracking of fish from the underwater videos. We benchmarked the performance of three OT architectures (MOSSE, Seq-NMS and SiamMask). To evaluate the accuracy of the OT architectures, we calculated precision, recall and a F1 score. We defined a true positive as a correct detection of all yellowfin bream and then accurate tracking of the individual for ≥ 50% of the time where yellowfin bream appeared on frame (Figure 1). A false negative was when a bream was not detected and tracked or if the yellowfin bream was tracked < 50% of the time when the fish appeared on frame (Figure 1). Additionally, we classified a false positive when a non-yellowfin bream object was detected and tracked or when a yellowfin bream was detected but the tracking architecture failed by then tracking a non-yellowfin bream object (Figure 1). When processing the tracking data, we grouped tracking angles using reference angles into four directions: up, down, right and left (Figure 2). Fish moving up meant that the fish movement had tracking angles between 44^o^ and 315^o^. Fish moving right had angles between 45^o^ and 135^o^, whereas fish moving left between 225^o^ and 315^o^. Finally, fish moving down had tracking angles between 135^o^ and 225^o^. The tracking angle for all OT architectures was obtained from the tracker vector that is generated within each tracker’s bounding box (Figure 2). By grouping the directions, we were able to count and group the number of movement angles per camera and per set (Figure 2). The net movement of a fish was measured as the direction (left, right, up or down) that had the highest proportion when the fish appeared on frame (Figure 2).


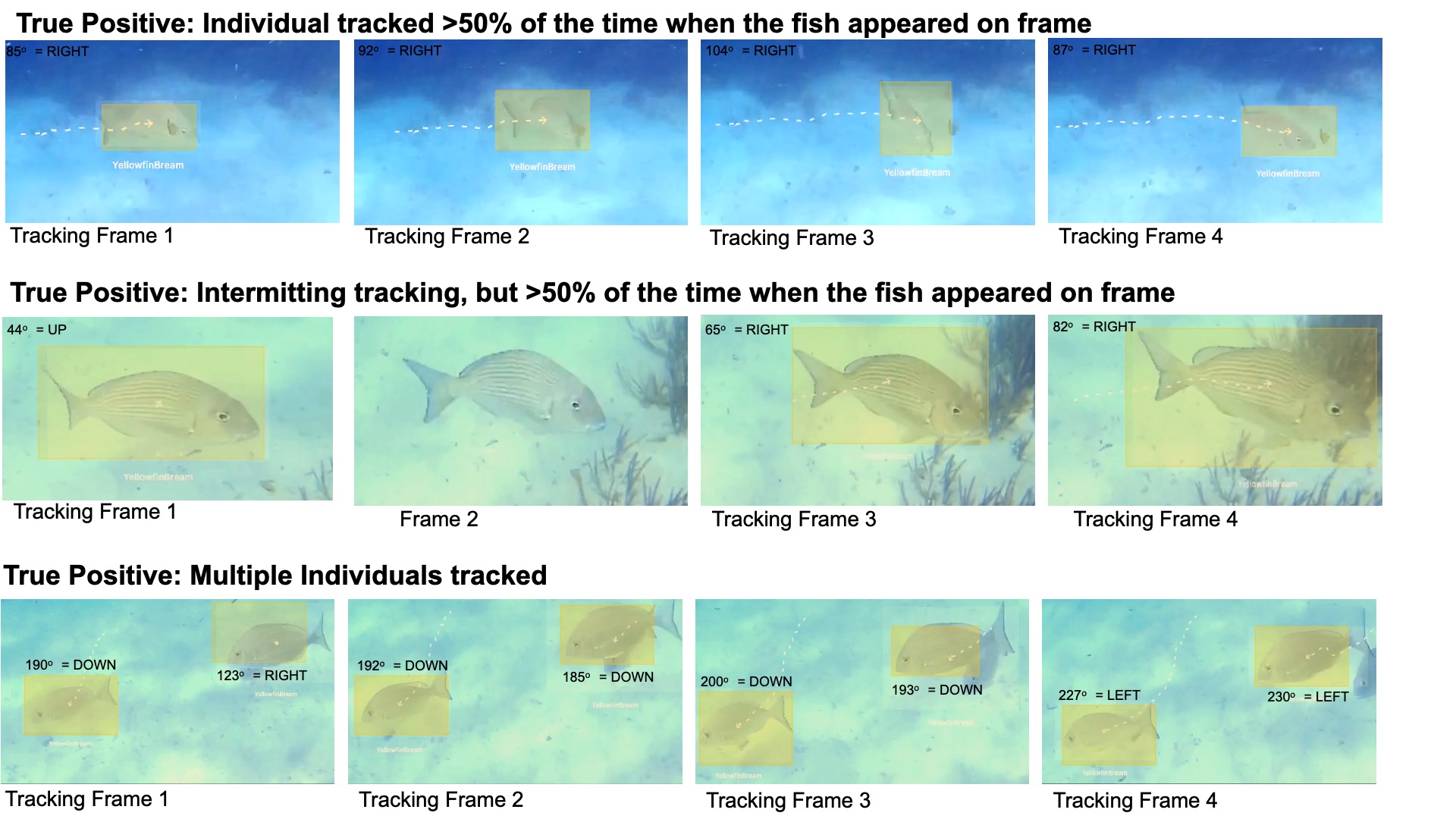


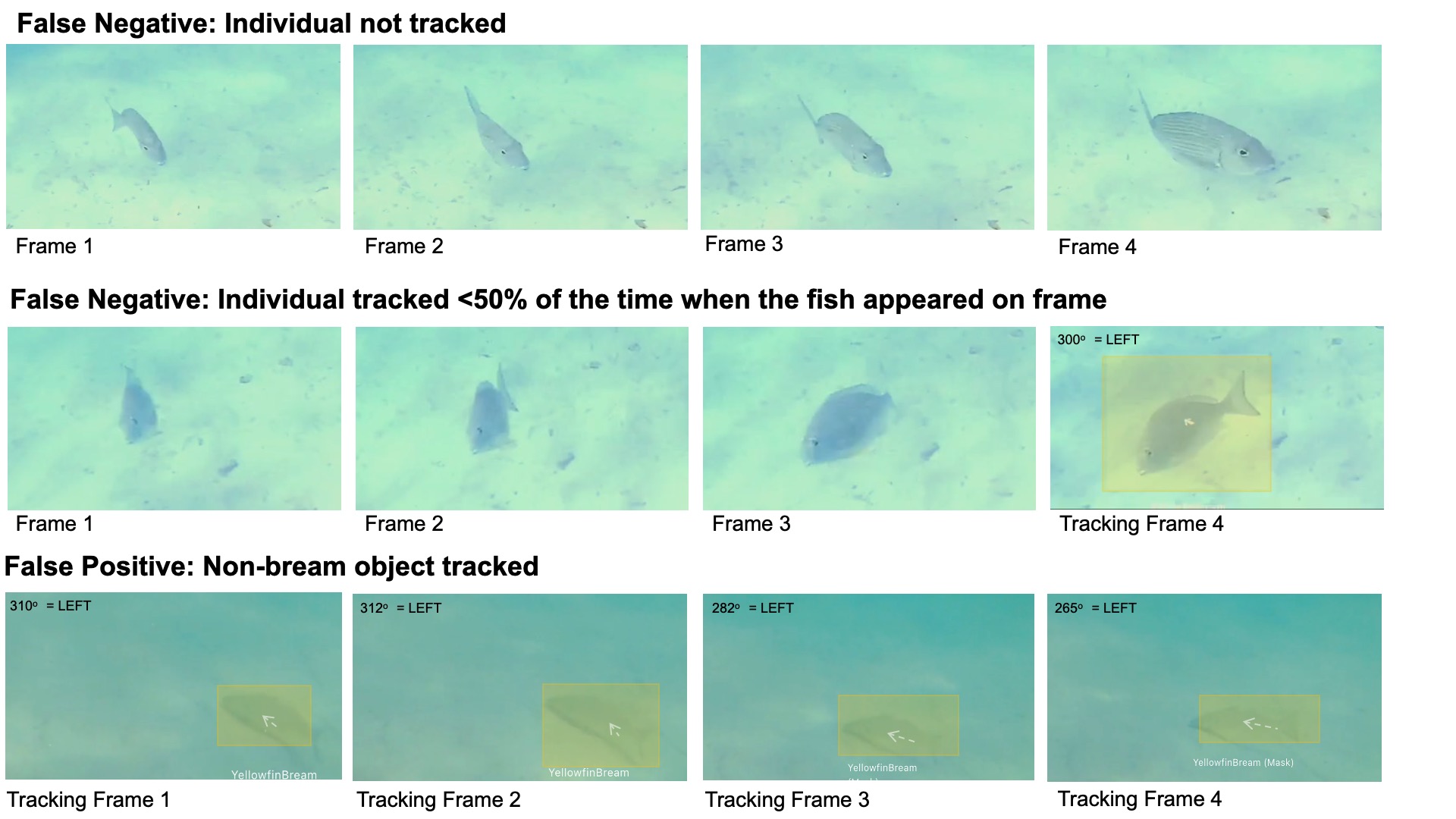


**FIGURE 1** Sample frames of videos processed through the computer vision pipeline. A true positive; a correct detection of all yellowfin bream and then accurate tracking of the individual for ≥ 50% of the time where yellowfin bream appeared on frame. A false negative; a failed bream detection and tracking or if the yellowfin bream was tracked < 50% of the time when the fish appeared on frame. Finally, a false positive; when a non-yellowfin bream object (in this case a sand whiting) was detected and tracked. Tracker bounding boxes displayed in yellow, tracker detection links display as white spotted lines and tracking angles with movement direction at top right corner of the frame. The object detection step was excluded from visualisation. Tracking visualisations shown for the highest performing object tracker – Seq-NMS.


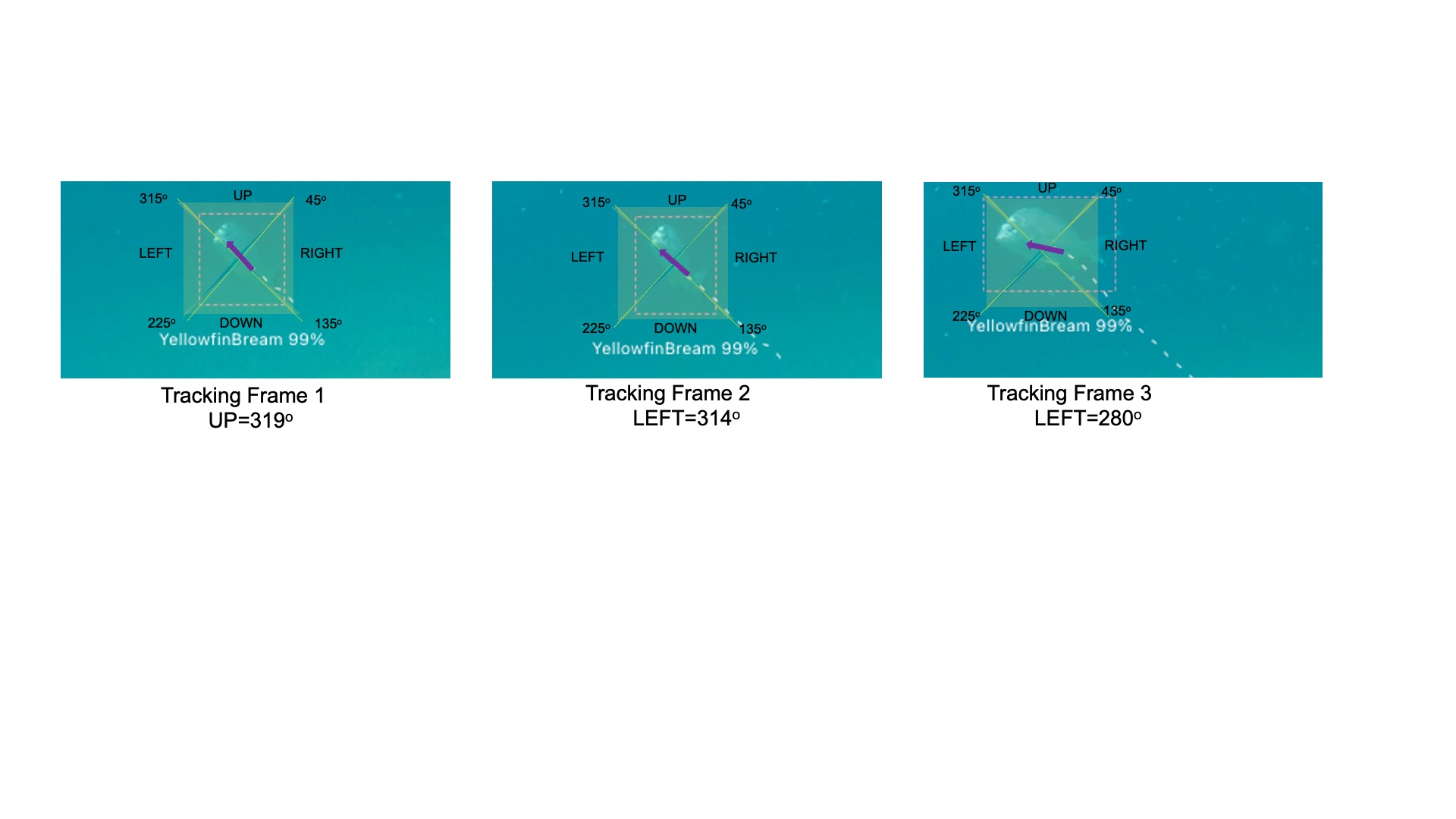


**FIGURE 2** Sample tracking visualisation of a yellowfin bream individual with the four references angles (45^o^, 135^o^, 225^o^ and 315^o^) used to group fish movement into four directions (up, down, left or right). 45^o^ divisions indicated as green lines, tracking vectors as a purple arrow in the middle of the fish and tracker boxes as red dotted boxes around the fish. The vector’s tracking angle indicates the movement of the fish. The net movement of a fish (white spotted line) was measured as the tracking direction with the highest proportion in the video; in this example the fish is moving left. The object detection step was excluded from visualisation; however, confidence of the detection is shown next to the label ‘YellowfinBream’.
